# Genome-Wide Landscape of mRNAs, lncRNAs, and circRNAs during Testicular Development of Yak

**DOI:** 10.3390/ijms24054420

**Published:** 2023-02-23

**Authors:** Yongfu La, Xiaoming Ma, Pengjia Bao, Min Chu, Ping Yan, Chunnian Liang, Xian Guo

**Affiliations:** 1 Animal Science Department, Lanzhou Institute of Husbandry and Pharmaceutical Sciences, Chinese Academy of Agricultural Sciences, Lanzhou 730050, China; 2 Key Laboratory of Animal Genetics and Breeding on Tibetan Plateau, Ministry of Agriculture and Rural Affairs, Chinese Academy of Agricultural Sciences, Lanzhou 730050, China; 3 Key Laboratory for Yak Genetics, Breeding, and Reproduction Engineering of Gansu Province, Chinese Academy of Agricultural Sciences, Lanzhou 730050, China

**Keywords:** testis, yak, development, non-coding RNA, reproduction

## Abstract

Testicular development is a tightly regulated process in mammals. Understanding the molecular mechanisms of yak testicular development will benefit the yak breeding industry. However, the roles of different RNAs, such as mRNA, lncRNA, and circRNA in the testicular development of yak, are still largely unclear. In this study, transcriptome analyses were performed on the expression profiles of mRNAs, lncRNAs, and circRNAs in testis tissues of Ashidan yak at different developmental stages, including 6-months-old (M6), 18-months-old (M18), and 30-months-old (M30). A total of 30, 23, and 277 common differentially expressed (DE) mRNAs, lncRNAs, and circRNAs were identified in M6, M18, and M30, respectively. Furthermore, functional enrichment analysis showed that the common DE mRNAs during the entire developmental process were mainly involved in gonadal mesoderm development, cell differentiation, and spermatogenesis processes. Additionally, co-expression network analysis identified the potential lncRNAs related to spermatogenesis, e.g., TCONS_00087394 and TCONS_00012202. Our study provides new information about changes in RNA expression during yak testicular development, which greatly improves our understanding of the molecular mechanisms regulating testicular development in yaks.

## 1. Introduction

The yak (*B. grunniens*) is an iconic symbol of the herbivore living on the “roof of the world”, found in and around the Himalayas and north at high altitudes of 2500 to 5500 m [1]. Yak production plays an important role in the Tibetan life of the people in high-altitude areas [2]. Survey reports reveal that the majority of yaks suffer from various reproductive problems like late maturity, long calving interval, poor oestrous expression, and repeat breeding [3]. Therefore, it is of practical significance to understand and master the reproductive and physiological characteristics of yaks. However, very little is known about molecular roles in the development and reproduction of Ashidan yaks, especially in yak testis.

Advances in science and technology have shown that mRNAs, lncRNAs, and circRNAs are involved in the regulation of biological processes in animals. Although lncRNAs were considered to be by-products of RNA polymerase II transcription and will not be translated into proteins, many lncRNAs are involved in nuclear transport, transcription activation and interference, and chromosomal and genome modification, thereby driving more researchers to explore how lncRNAs affect human biology [4,5]. Many lncRNAs were identified from testis at different developmental stages, and they were predicted to play key roles in testis development and spermatogenesis in rats, mice, and cattle [6,7,8]. Currently, there were few studies on the effect of lncRNAs on testicular development and spermatogenesis. For example, LOC102176306 and miR-1197-3p regulate testosterone production and cell proliferation by regulating PPARGC1A expression in goat Leydig cells [9]. Although spermatogenesis and testicular development are partially regulated through the action of lncRNAs, the functions of most of them have not been determined.

CircRNA biosynthesis is regulated by cis- and trans-factors, and its expression is tissue and cell-specific [10]. Some circRNAs are evolutionarily conserved, and they play important biological functions as miRNA inhibitors or by regulating protein function. CircRNAs play key roles in cell migration, proliferation, and differentiation [11]. To date, there were few studies on the expression patterns of lncRNAs and circRNAs in yak testis. To investigate the effect of ncRNAs on yak testis development, we performed transcriptome analysis on the expression profiles of mRNAs, lncRNAs, and circRNAs in yak testis tissues at different developmental stages. Our study will provide a good model for studying the mechanisms that regulate testicular development and spermatogenesis and provides newer insights regarding the regulation of male yak reproduction.

## 2. Results

### 2.1. Overview of the Sequencing Data

A total of 274.25, 285.88, and 299.67 M clean reads were obtained in M6, M18, and M30, respectively. The Q30 values were 94.68–95.11% while the GC content ranged between 46.47% and 48.87%. About 94% of the clean reads were successfully mapped in the yak genome. Of the successfully mapped reads, 83.69% of the uniquely mapped reads were used for transcript construction (Table 1).

### 2.2. Predictions and Properties of ncRNAs in the Yak Testis

**Figure 1:**
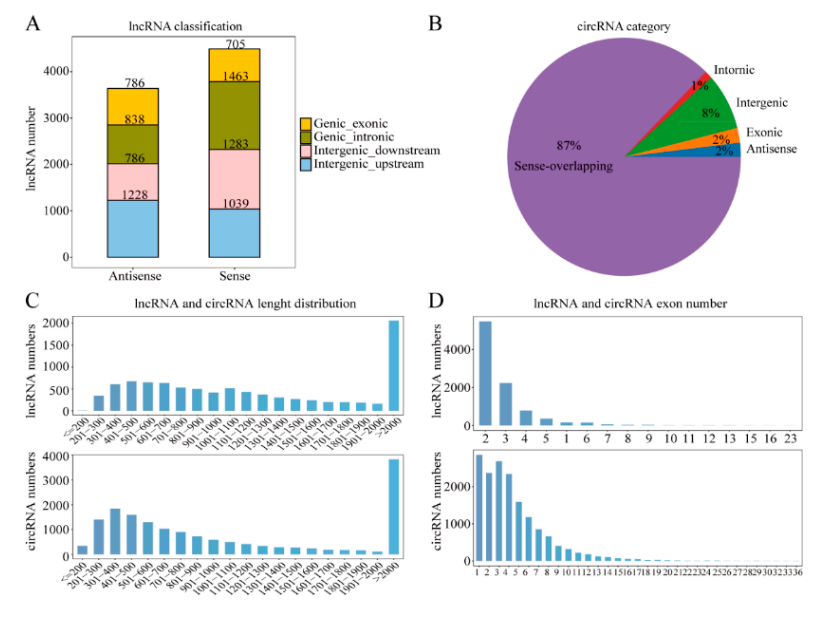
Gene expression characterization. (**A**) The positional relationship between lncRNA and known protein-encoded transcripts counts lncRNA types from four aspects: direction, type, location, and subtype. (**B**) The percentage of five types of circRNAs. (**C**) The length distribution of lncRNAs and circRNAs. (**D**) The number of exons per lncRNA and circRNAs.

A total of 20,953 mRNAs, 10,591 lncRNAs, and 16,185 circRNAs were identified from the 6, 18, and 30-month yak testis. Through genome alignment, these lncRNAs transcripts were classified into exon antisense, intron antisense, intergene downstream antisense, intergene upstream antisense, exonic sense, intron sense, intergene downstream sense, and intergene upstream sense, including 786, 838, 786, 1228, 705, 1463, 1283 and 1039, respectively (Figure 1A). However, sense overlapping circRNAs for 87.35%, intergene circRNAs for 8%, and the least located in introns (Figure 1B). Most lncRNAs with lengths greater than 2000 bp contain two exons (Figure 1C). Similarly, most circRNAs are longer than 2000 bp and contain less than 6 exons. (Figure 1D).

### 2.3. Differential Expression Analysis of mRNAs, lncRNAs, and circRNA

**Figure 2:**
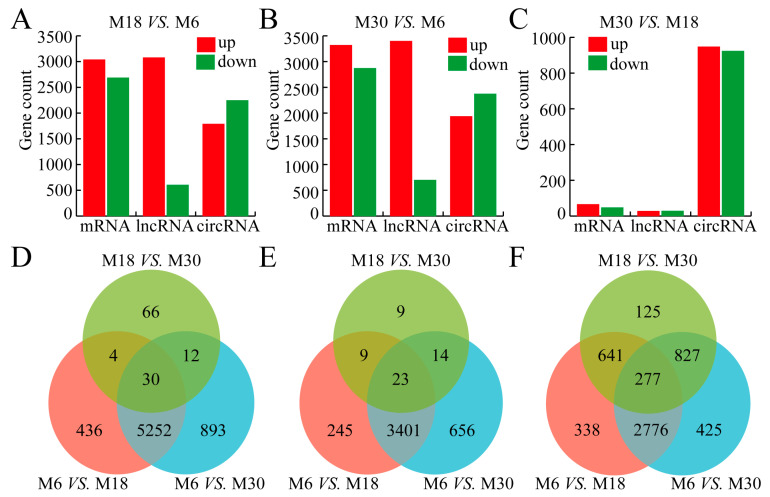
Comparative analysis of mRNAs, lncRNAs, and circRNAs in different developmental stages of yak testis. (**A**) Histogram representing the numbers of upregulated and downregulated mRNAs and ncRNAs between 18 and 6-month groups. (**B**) Histogram representing the numbers of upregulated and downregulated mRNAs and ncRNAs between 30 and 6-month groups. (**C**) Histogram representing the numbers of upregulated and downregulated mRNAs and ncRNAs between 30 and 18-month groups. (**D**) The number of common DE mRNAs. (**E**) The number of common DE lncRNAs. (**F**) The number of common DE circRNAs.

To evaluate the differences in gene expression patterns during three developmental stages, we performed pairwise comparisons between the three developmental stages. For M18 vs. M6, 3037 mRNAs, 3076 lncRNAs, and 1787 circRNAs were upregulated, 2685 mRNAs, 602 lncRNAs, and 2245 circRNAs were downregulated (Figure 2A and Tables S1–S3). For M30 vs. M6, 3317 mRNAs, 3397 lncRNAs, and 1934 circRNAs were upregulated, 2870 mRNAs, 697 lncRNAs, and 2371 circRNAs were downregulated (Figure 2B and Tables S4–S6). For M30 vs. M18, 65 mRNAs, 27 lncRNAs, and 947 circRNAs were upregulated, 47 mRNAs, 28 lncRNAs, and 923 circRNAs were downregulated (Figure 2C and Tables S7–S9). There were 30 mRNAs, 23 lncRNAs, and 277 circRNAs were differentially expressed in all three developmental stages (Figure 2D–F and Table S10).

### 2.4. Gene ontology (GO) Analysis of DE Genes between M30 and M6

**Figure 3:**
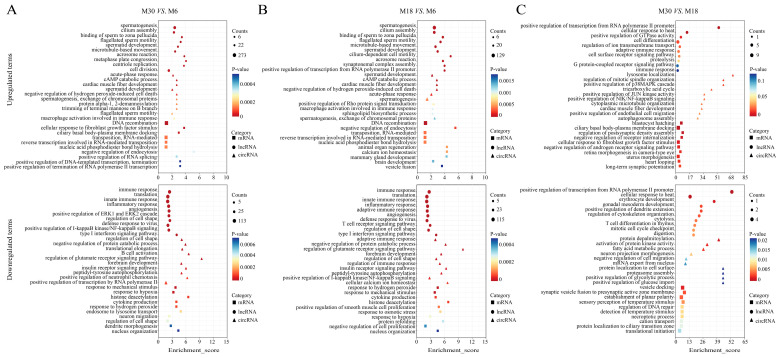
GO analysis of DE genes (including mRNAs, lncRNAs, and circRNAs) in the M30 vs. M6, M18 vs. M6, and M30 vs. M18 groups. (**A**) GO analysis of DE genes in the M30 vs. M6 group. (**B**) GO analysis of DE genes in the M18 vs. M6 group. (**C**) GO analysis of DE genes in the M30 vs. M18 group. The top 10 enriched GO terms ranked by *p*-values are shown (*p*-value < 0.05).

Between M30 and M6, significantly up-regulated GO terms were mainly involved in the spermatogenesis, cell division, and spermatid development for DE mRNAs and DE lncRNAs, and DNA recombination, positive regulation of DNA-templated transcription, termination, cellular response to fibroblast growth factor stimulus and transposition, RNA-mediated for DE circRNAs (Figure 3A and Table S11). Furthermore, significantly down-regulated GO terms were mainly involved in the angiogenesis, regulation of cell shape, and positive regulation of cell migration for DE mRNAs, DE lncRNAs, and DE circRNAs (Figure 3A and Table S12).

### 2.5. Gene Ontology (GO) Analysis of DE Genes between M18 and M6

Between M18 and M6, significantly up-regulated GO terms were mainly involved in the spermatogenesis, spermatid development for DE mRNAs and DE lncRNAs, and DNA recombination, negative regulation of endocytosis and animal organ regeneration for DE circRNAs (Figure 3B and Table S13). Furthermore, significantly down-regulated GO terms were mainly involved in the angiogenesis, regulation of cell shape for DE mRNAs, and regulation of cell shape and maintenance of DNA methylation for DE lncRNAs, and cytokine production negative regulation of cell proliferation for DE circRNAs (Figure 3B and Table S14).

### 2.6. Gene Ontology (GO) Analysis of DE Genes between M30 and M18

Between M30 and M18, significantly up-regulated GO terms were mainly involved in the negative regulation of endopeptidase activity and G protein-coupled receptor signaling pathway for DE mRNAs, and spermatid development, cell differentiation, DNA recombination for DE lncRNAs, and negative regulation of androgen receptor signaling pathway and cellular response to fibroblast growth factor stimulus for DE circRNAs (Figure 3C and Table S15). Furthermore, significantly down-regulated GO terms were mainly involved in the cell cycle, positive regulation of cell proliferation for DE mRNAs, protein ubiquitination and DNA recombination for DE lncRNAs, and regulation of DNA repair and transnational initiation for DE circRNAs (Figure 3C and Table S16).

### 2.7. Function Analysis of Common DE Genes during Testicular Development

**Figure 4:**
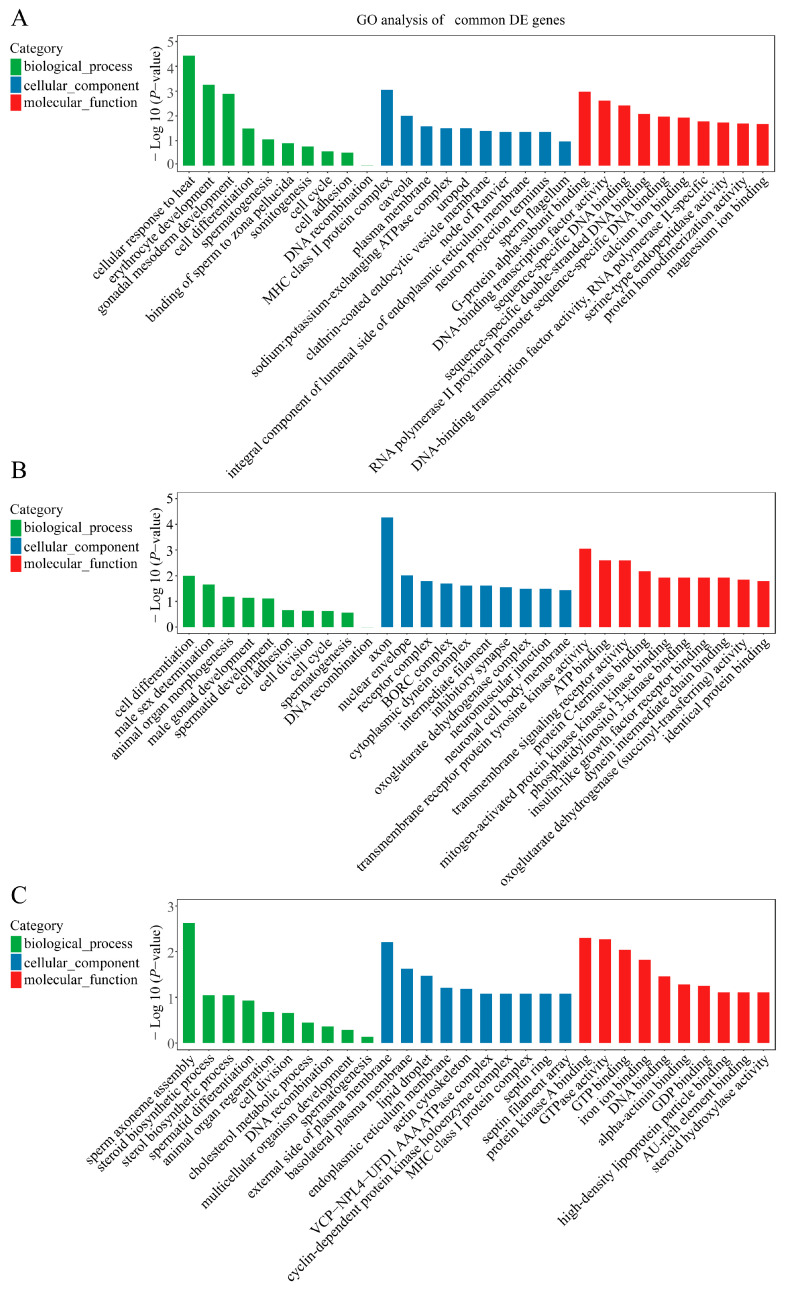
GO analysis of common DE genes. (**A**) GO analysis of common DE mRNAs. (**B**) GO analysis of common DE lncRNAs. (**C**) GO analysis of common DE circRNAs. The top 10 enriched GO terms ranked by *p*-values are shown (*p*-value < 0.05).

Compared with 6 months of age, 30 mRNAs, 23 lncRNAs, and 277 circRNAs were identified as common DE genes during testicular development. GO analysis showed that the terms were mainly involved in the erythrocyte development, gonadal mesoderm development, cell differentiation and spermatogenesis for DE mRNAs (Figure 4A and Table S17), and negative regulation of signal transduction, cell differentiation, and positive regulation of meiotic cell cycle for DE lncRNAs (Figure 4B and Table S17), and sperm axoneme assembly, positive regulation of secretion, positive regulation of cell cycle and positive regulation of cell proliferation for DE circRNAs (Figure 4C and Table S17).

**Figure 5:**
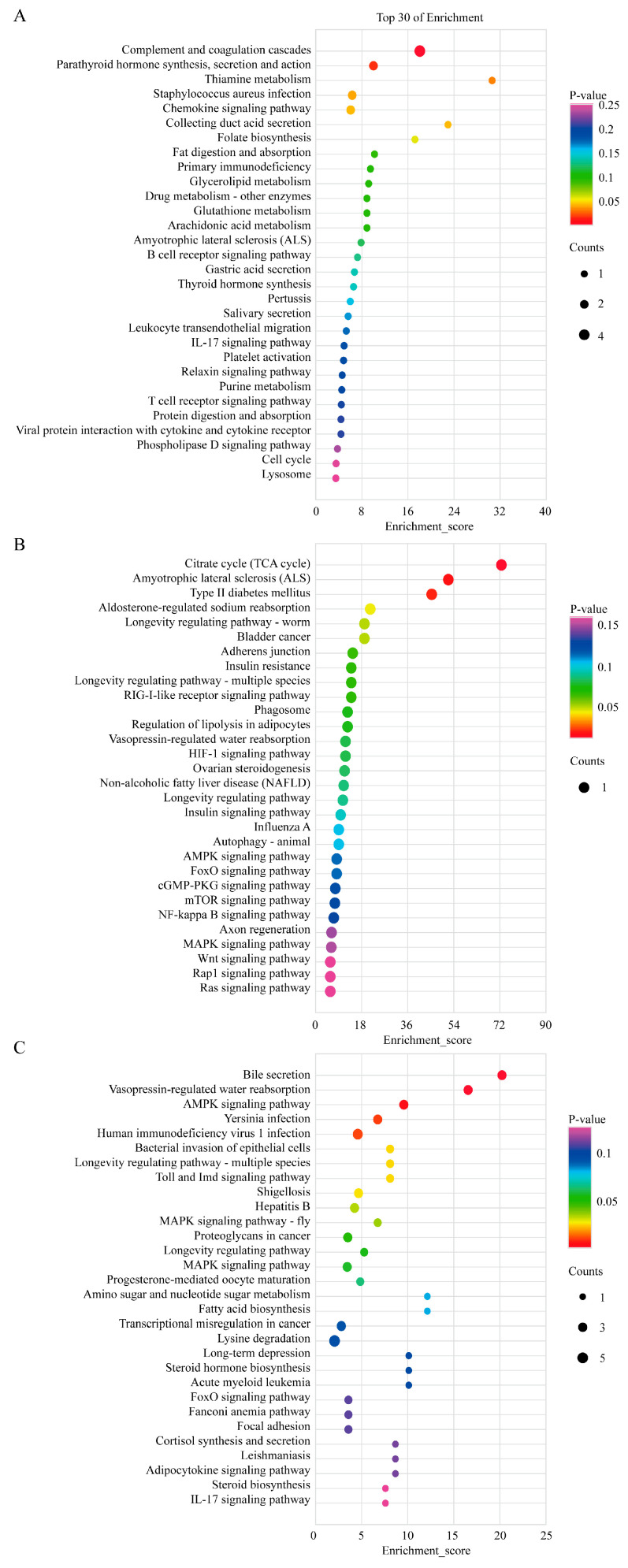
KEGG analysis of common DE genes. (**A**) KEGG analysis of common DE mRNAs. (**B**) KEGG analysis of common DE lncRNAs. (**C**) KEGG analysis of common DE circRNAs. The top 30 enriched KEGG terms ranked by *p*-values are shown (*p*-value < 0.05).

The top 30 KEGG pathways are shown in Figure 5. KEGG analysis of the common DE mRNAs indicated that these genes were involved in the PI3K-Akt signaling pathway, parathyroid hormone synthesis, secretion and action, chemokine signaling pathway, and thyroid hormone synthesis (Figure 5A). KEGG analysis of the common DE lncRNAs indicated that target genes were involved in the AMPK signaling pathway, wnt signaling pathway, aldosterone-regulated sodium reabsorption, and PI3K-Akt signaling pathway (Figure 5B). Similarly, the common DE circRNAs parent genes were associated with the AMPK signaling pathway, MAPK signaling pathway, steroid hormone biosynthesis, FoxO signaling pathway, adipocytokine signaling pathway, and steroid biosynthesis (Figure 5C).

### 2.8. Construction of the lncRNA-mRNA Co-Expression Network

**Figure 6:**
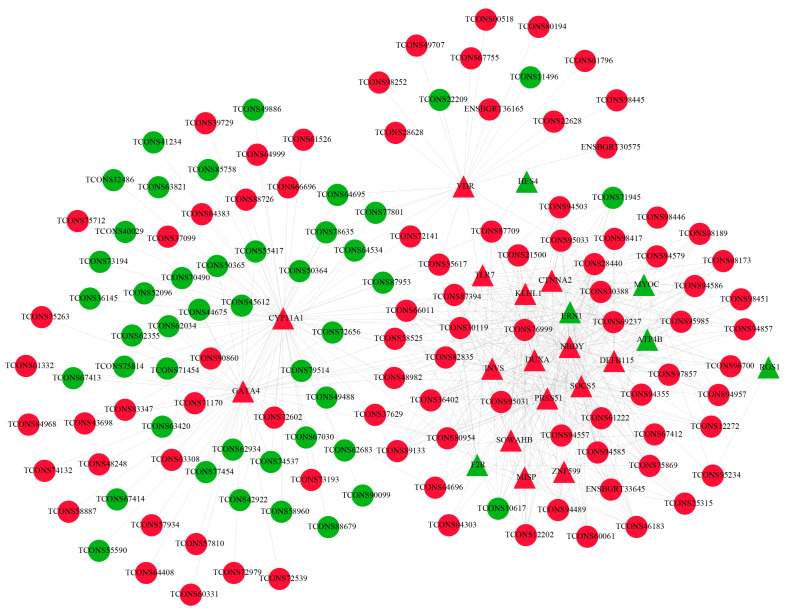
Construction of the lncRNA-mRNA co-expression network. Co-expressed network of common DE lncRNAs and their targeted common DE mRNAs involved in spermatogenesis and cell differentiation processes. Red and green represent upregulated and downregulated, respectively. Ellipses and triangles represent lncRNAs and mRNAs, respectively.

To better understand the relationship between male reproduction and testicular development, 21 common DE mRNAs related to spermatogenesis and cell differentiation processes and 22 common DE lncRNAs targeting them were selected to construct the mRNA-lncRNA co-expression network. The results showed that the co-expression network comprised 255 connections and each lncRNA may be related to multiple mRNAs (Figure 6). Noticeably, DE lncRNAs TCONS_00087394, TCONS_00066011, and TCONS_00012202 were involved in the regulation of several DE mRNAs such as INVS, TLR7, PRSS51, F2R, and NBDY, indicating that these lncRNAs might play an important role in regulating spermatogenesis and cell differentiation processes.

### 2.9. Validation of DE Genes by qRT-PCR

**Figure 7:**
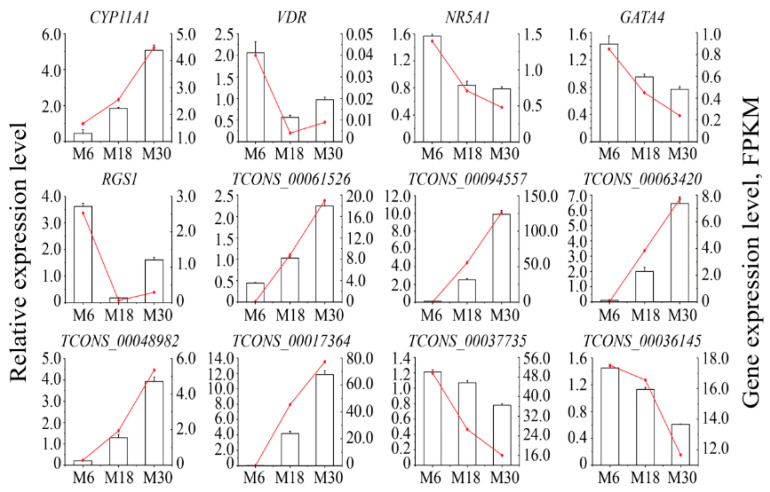
Validation of the expression of differentially expressed genes by qRT-PCR. Data from the qRT-PCR are shown as columns and on the *Y*-axis on the left, while the data from RNA-Seq are shown as lines and on the *Y*-axis on the right.

A total of twelve genes, including five mRNAs (CYP11A1, VDR, NR5A1, GATA4, and RGS1) related to testicular development, and Leydig cells growth and development and seven random lncRNAs (TCONS_00061526, TCONS_00094557, TCONS_00063420, TCONS_00048982, TCONS_00017364, TCONS_00037735, and TCONS_00036145) for qRT-PCR verification. The comparison found that the expression trends of RNA-Seq and qRT-PCR were similar, which confirmed the reliability of the sequencing data (Figure 7).

## 3. Discussion

The main function of the testis is to produce sperm and synthesize hormones. Testicular development and spermatogenesis are tightly regulated process that primarily involves the localization and differentiation of Leydig cells, Sertoli cells, and germ cells, requiring precise control of numerous genes and networks that act synergistically or antagonistically at the transcription and post-transcription levels [12]. Therefore, it is very important to determine the regulatory mechanism of testicular development and spermatogenesis for the study of yak breeding. In particular, the regulatory roles of lncRNAs and circRNAs on target genes have been extensively studied in organ development. In our study, we investigated the mRNAs, lncRNAs, and circRNAs expression profiles of 6, 18, and 30 months yak testis, which included juvenile, pubertal, and sexual maturation of testicular development [13]. Although previous studies have shown that lncRNAs and circRNAs are involved in testis development, the dynamic process of expression profiles of lncRNAs and circRNAs in yak testis is rare, and our study provides a theoretical basis for future new explorations.

Normal testicular development and spermatogenesis are the basis for ensuring the reproductive ability of male animals, which are regulated by different genes or different expression levels of the same gene at different developmental stages [14]. LncRNAs and circRNAs have received increasing attention as the most popular ncRNAs, which participate in the regulation of different biological processes in different ways [15,16]. LncRNA has become a major regulatory factor in animal reproductive processes such as sex hormone responses, gonadogenesis, spermatogenesis, sex determination, and meiosis [17]. In this study, 6-, 18- and 30-month-olds correspond to infant, adolescent, and adult stages in male yaks presenting the dynamic process of testis development. A total of 5722, 6187, and 112 DE mRNAs, 3678, 4094, and 55 DE lncRNAs, and 4032, 4305, and 1870 DE circRNAs were identified between M18 and M6, between M30 and M6, and between M30 and M18, respectively. Only 30 DE mRNAs, 23 DE lncRNAs, and 277 DE circRNAs were identified from the testis of yak at the age of 6, 18, and 30 months old. At the same time, we discovered many novel lncRNAs and circRNAs during the research process, which may indicate that compared with other animals, the research on lncRNAs and circRNAs in yak testis tissue is still limited. Since the novel lncRNAs and circRNAs were obtained through the yak genome alignment and their characteristics, and the identification criteria were strict, they are of high value for future research on the molecular mechanisms of male yak testis development and spermatogenesis. Furthermore, lncRNAs and circRNAs have lowered lengths, exon numbers, and expression levels compared to mRNAs. The characteristics and differences of lncRNAs and circRNAs have also been found in other animals, which may suggest that there is also some conservation in the regulation of lncRNAs and circRNAs in mammals [18]. We selected five mRNAs and seven lncRNAs related to testis development, Leydig cell growth, and development for qRT-PCR to validate the accuracy of RNA-seq data. Through analysis, it was found that qRT-PCR and RNA-Seq data have similar expression trends, indicating that our RNA-Seq data is of high quality and sequencing data can be used for in-depth analysis.

Generally speaking, the main function of the testis is to produce sperm and androgen, which depends on the normal development of both testicular somatic cells and germ cells. They first guide fetal germ cell differentiation toward spermatogenic destiny and then take care of the full service to spermatogenic cells during spermatogenesis. The number of Sertoli cells sets the limits of sperm production. Leydig cells secrete androgens that determine masculine development. Testis development does not depend on germ cells, testicular somatic cells also develop in the absence of germ cells, but spermatogenic cell development is dependent on somatic cells [19]. In this study, we performed GO enrichment analyses that can help to elucidate the functions and pathways involved in candidate target genes. Our results showed that differentially expressed RNAs in 6-, 18- and 30-month-old testis were mostly enriched in GO terms related to spermatogenesis, cell proliferation, positive regulation of cell proliferation, and cell differentiation. At the same time, more differentially expressed RNAs were observed in the M30 vs. M6 and M18 vs. M6 comparison groups compared with M30 vs. M18, suggesting that more RNAs were involved in cell differentiation and proliferation processes in the early stages of testis development while indicating that Cell proliferation and differentiation in the testis of early animals is more vigorous. However, further studies are needed to confirm this speculation.

Based on the above studies, we suggested that the transcription differences were caused by changes in cell types in the yak testis during testicular development and that lncRNAs might play important regulatory roles in male yak sexual maturation. Previous studies have demonstrated that certain lncRNAs play important regulatory roles during testicular development and spermatogenesis in male animals [20,21]. In this study, the differentially expressed lncRNAs TCONS_00061526, TCONS_00094557, TCONS_00063420, TCONS_00048982, TCONS_00017364, TCONS_00037735 and TCONS_00036145 targeted to regulate the expression levels of CYP11A1, VDR, NR5A1, GATA4, and RGS1 genes, and GO analysis found that these genes were enriched in biological processes such as gonadal mesoderm development, cell differentiation, spermatogenesis, cell cycle, and DNA recombination. The CYP11A1 gene encodes the CYP11A1 enzyme, which is located in the inner mitochondrial membrane and catalyzes the conversion of cholesterol to pregnenolone in the first and rate-limiting step of the steroid hormone synthesis [22]. Studies of the CYP11A1 gene suggested that this gene plays an important role in gene regulation, testosterone secretion, and male reproductive organ development [23]. Similarly, in this study, the CYP11A1 gene was significantly upregulated in the 30-month-old group compared with the 6-month-old, suggesting that the CYP11A1 gene might regulate the yak testis development by promoting steroid hormone synthesis. GATA4 is expressed in Leydig and Sertoli cells, is required for mouse fetal testis development, and is a key transcription regulator of Sertoli cell function in adult mice [24]. Studies of chimeric mice derived from Gata4-/- embryonic stem cells show that GATA4 plays an integral role in the development of fetal Leydig cells [25]. In this study, GATA4 has the highest expression in the testis of sexually mature yak, and we speculated that this gene might promote spermatogenesis by regulating the functions of the Leydig and Sertoli cells, thereby achieving precocious puberty. Furthermore, the constructed lncRNA-mRNA co-expression network shows that lncRNAs (such as TCONS_00087394, TCONS_00066011, and TCONS_00012202) were regulated by multiple lncRNAs, suggesting that these lncRNAs and their target mRNAs might also play an important role in yak testicular development. However, the underlying mechanisms of these interacting lncRNA and mRNA regulatory activities need to be further investigated.

## 4. Materials and Methods 

### 4.1. Ethics Statement

All yaks were handled in strict accordance with the Animal Ethics Procedures and Guidelines of the People’s Republic of China. The present study was approved by the Animal Administration and Ethics Committee of the Lanzhou Institute of Husbandry and Pharmaceutical Sciences of the Chinese Academy of Agricultural Sciences (Permit No. 2019-002).

### 4.2. Animals

All animals used in this study were from the Datong Breeding Farm of Qinghai province. The nine selected Ashidan yak were healthy and fed in similar conditions. Furthermore, the animals were separated into three groups (6 months, M6; 18 months, M18; and 30 months, M30). Every group contained three male yaks. The nine male yaks were slaughtered and tissues from the left testis were collected. All samples were immediately stored at −80 °C for total RNA extraction.

### 4.3. RNA-Sequencing Data Analysis

Total RNA was extracted from testis tissue using TRIzol (Invitrogen, Carlsbad, CA, USA). Genomics DNA was removed using DNase I. A total of 9 cDNA libraries were constructed in this study using the NEB Next Ultra Directional RNA LibraryPrep Kit for Illumina (NEB, Ipswich, MA, USA). Sequencing libraries were then sequenced on an Illumina HiseqTM 2500 (Illumina Corp., San Diego, CA, USA) instrument to generate 150-nt paired-end reads. Libraries were constructed and sequenced using the Illumina platform by OE Biotech Co. (Shanghai, China). Then, the raw data was checked using the FASTQC (version 0.11.9) tools. Trimmomatic (version 0.36) software was first used for removing adapters, and then low-quality bases and N-bases or low-quality reads were filtered out. The quality of the trimmed reads was rechecked with the FASTQC tool, after which the clean reads were aligned with the yak genome using HISAT2 (version 2.0.5) [26]. The genome sequence of yak (BosGru_v3.0) and the annotation file was downloaded from Ensemble. Transcripts were assembled by Stringtie (version 1.3.4) [27]. Raw data for RNA-seq has been documented in the SRA public database (Accession number: SRP367128).

### 4.4. Identification of lncRNA and circRNA

Use the following procedure to identify lncRNAs: (1) Transcripts annotated as “i (a transfrag falling entirely within a reference intron)”, “u (unknown, intergenic transcript)”, “x (exonic overlap with reference on the opposite strand)” and “o (generic exonic overlap with a reference transcript)” were retained by the cuffcompare software [28]. (2) Transcripts longer than 200 bp and containing more than 2 exons were retained. (3) Finally, four approaches of Coding-Non-Coding-Index (CNCI, score < 0), Coding Potential Calculator (CPC, score < 0), Pfam (E value < 0.001), and k-mer scheme (PLEK, score < 0) were used to predict coding potential, and transcripts without coding potential were candidate lncRNAs [15]. Then, CIRI software was used to scan for PCC signals (paired chiastic clipping signals), and circRNA sequences were predicted based on junction reads and GT-AG cleavage signals [29]. Briefly, paired chiastic clipping, paired-end mapping, and GT-AG splicing signals were found by scanning the obtained slicing alignments. Next, the alignment files were scanned again using a dynamic programming algorithm to detect additional junction reads and eliminate false-positive circRNA candidates. Final circRNAs were obtained by retaining sequences with ≥2 junction reads.

### 4.5. Analysis of Differentially Expressed (DE) Genes

The fragments per kilobase of transcript per million read mapped (FPKM) and spliced reads per billion mappings (SRPBM) values were used to detect the expression levels of mRNA, lncRNA, and circRNA, respectively [30]. Differentially expressed mRNAs, lncRNAs, and circRNAs were detected using the DESeq2 software package [31], and differentially expressed genes were defined as |log2 (fold change)| ≥ 1 and FDR < 0.001 between any comparison groups. Meanwhile, genes differentially expressed in three comparisons (M6 vs. M18, M6 vs. M30, and M18 vs. M30) were defined as common DE genes.

### 4.6. Functional Enrichment Analysis

The cis-target mRNAs were screened by the genomics location 50 Kb upstream and downstream of the lncRNA, trans-target mRNAs were identified by the Pearson correlation coefficient of the lncRNA-RNA pairs being ≥ 0.9, and then the cis- and trans-target mRNAs were subjected to GO analysis. The parental genes of DE circRNAs were mapped to GO terms using the Gene Ontology database (http://www.geneontology.org) (accessed on 14 May 2022), followed by enrichment analysis with the Omicshare (version 3.0) tools. Kyoto Encyclopedia of Genes and Genomes (KEGG) analysis was also performed to annotate the signaling pathways associated with these common DE genes [32]. Terms with *p*-values less than 0.05 were identified as significant or enriched terms.

### 4.7. Co-Expression Network Construction

To study the functions of key lncRNAs, Cytoscape software (version 3.1.1) was used to construct the co-expression network of common DE lncRNAs and common DE mRNAs [33].

### 4.8. Gene Expression Validation by Quantitative Real-Time PCR

We used qRT-PCR to verify the gene expression levels. The PCR reaction was performed on the LightCycler 480 II (Roche, Basel, Switzerland) using the SYBR Green Real-time PCR Master Mix (TOYOBOCO, Ltd., Osaka, Japan) with different cycling conditions as 95 °C for 10 min, followed by 45 cycles for 15 s at 95 °C, annealing for 60 s at 55 to 60 °C, extension for 30 s at 72 °C, final extension for 5 min at 72 °C, and storage at 4 °C. GAPDH was used as an internal reference to normalize target gene expression. All experiments were performed in triplicate. The primers (Table S18) were produced by Sheng gong Biotech Co., Ltd. (Shanghai, China). For gene expression levels, each experiment was repeated in at least 3 replicates, and the threshold cycles were calculated using the 2^-ΔΔCt^ method [34]. All data were expressed as “means ± SD”. A *p*-value < 0.05 was established as a significant difference.

## 5. Conclusions

In conclusion, a genome-wide view of the mRNAs, lncRNAs, and circRNAs expression profiles during yak testis development was explored in our study. In addition, we identified several DE genes that may contribute to testicular development and spermatogenesis. Our study provides a comprehensive basis for the expression levels of mRNAs, lncRNAs, and circRNAs during the yak testicular development, thus providing new clues for our understanding of the molecular regulatory mechanism of yak testis development.

## Figures and Tables

**Table 1:** Summary of RNA-Seq data and mapping.

Sample	Raw Reads	Clean Reads	Q30 (%)	GC Content (%)	Mapped Reads	Uniquely Mapped
M18-1	101.07M	99.83M	95.11	48.28	89.95M (94.48%)	80.21M (84.26%)
M18-2	91.07M	89.98M	95.09	48.33	81.17M (94.60%)	72.12M (84.05%)
M18-4	97.24M	96.07M	95.14	48.87	86.66M (94.59%)	76.68M (83.69%)
M6-2	88.87M	87.66M	94.89	47.93	78.59M (94.01%)	70.38M (84.19%)
M6-3	91.57M	90.39M	94.83	46.47	81.29M (94.31%)	74.12M (85.98%)
M6-4	97.52M	96.20M	94.68	47.49	86.61M (94.41%)	78.23M (85.27%)
M30-1	100.51M	99.26M	94.96	48.20	89.58M (94.63%)	79.83M (84.34%)
M30-2	103.01M	101.81M	95.06	48.13	92.03M (94.78%)	81.81M (84.25%)
M30-3	99.81M	98.60M	95.05	48.06	89.03M (94.67%)	79.24M (84.27%)

## Data Availability

The data presented in this study are openly available in the SRA public database (Accession number: SRP367128).

## Supplementary Materials

The following supporting information can be downloaded at: https://www.mdpi.com/article/10.3390/ijms24054420/s1.

## Abbreviations

DE	differentially expressed
GO	Gene ontology
KEGG	Kyoto Encyclopedia of Genes and Genomes
qRT-PCR	Quantitative real-time PCR
M6	6-month-old
M18	18-month-old
M30	30-month-old
FPKM	fragments per kilobase of transcript per million reads mapped
SRPBM	spliced reads per billion mappings
LncRNAs	long non-coding RNAs
CircRNAs	circular RNAs
